# PW03-024 - A transgenic mouse model for variant procaspase-1

**DOI:** 10.1186/1546-0096-11-S1-A250

**Published:** 2013-11-08

**Authors:** A Hermsdorf, F Pessler, H Luksch, S Winkler, R Naumann, J Roesler, A Roers, A Rösen-Wolff

**Affiliations:** 1 Department of Pediatrics, University Clinic Carl Gustav Carus, TU Dresden, Dresden, Germany; 2 TWINCORE Center for Clinical and Experimental Infection Research, Hannover, Germany; 3 Max Planck Institute of Molecular Cell Biology and Genetics, Germany; 4 Insitute of Immunology, TU Dresden, Dresden, Germany

## Introduction

We have detected several genetic variants of *CASP1* in patients suffering from unexplained recurrent febrile episodes. Paradoxically, in vitro and in vivo analyses of patients’ cells revealed decreased enzymatic activity of these caspase-1 variants leading to impaired cytokine production despite the proinflammatory phenotype of the patients. The pathophysiological processes associated with *CASP1* variants are still under investigation.

## Objectives

In order to recapitulate the effects of the *CASP1* mutations found in the patients we tried to establish a bacterial artificial chromosome (BAC) transgenic mouse line expressing enzymatically inactive *Casp1^C284A^* under the control of the own promoter.

## Methods

The purified BAC fragment containing Flag-tagged *Casp1*^C284A^ (*Casp1*^C284AFlag^) was injected into the pronuclei of fertilized C57Bl6 mouse oocytes, followed by transfer of these oocytes to pseudopregnant foster mothers. Pups born from these mothers were analyzed for the presence of full-length *Casp1*^C284AFlag^ by screening with sequence specific PCR, Southern blot, and sequencing of the transgene. *Casp1*^C284AFlag^ transgenic mice were crossed to conventional *Casp1* knock-out (KO) mice and the immunological phenotype of the progeny was analyzed by in vitro stimulation of BMDCs. Expression levels of the *Casp1*^C284AFlag^ transgene were quantified by qRT-PCR and Western blots. Released cytokine levels were determined by cytometric bead arrays.

## Results

From two independent pronucleus injections we received 180 pups. Only three of them harbored transgene sequences and only one female animal proved to harbor the complete *Casp1^C284AFlag^* transgene (TG). Crossing to *Casp1* KO mice yielded the following genotypes: *Casp1*WT/WT/TG, *Casp1*WT/KO/TG, and *Casp1*KO/KO/TG. qRT-PCR analyses revealed that unstimulated *Casp1^C284AFlag^* transcription was reduced to 0.1% of wild-type *Casp1*. Hence, protein expression could not be detected in unstimulated cells. However, stimulation with LPS upregulated transcription and low-level translation of Casp1^C284AFlag^ in BMDCs. Determination of released cytokines after LPS/ATP stimulation revealed increased release of IL-6 and TNF-a from *Casp1*WT/KO/TG mice with proven Casp1^C284AFlag^ expression.

## Conclusion

These data indicate that even tiny amounts of Casp1^C284AFlag^ induced release of other proinflammatory cytokines and that this might contribute to the proinflammatory phenotype observed in our patients. Baseline expression of enzymatically inactive Casp1^C284AFlag^ may be embryonically lethal in mice since not a single mouse could be generated which expressed the transgene under unstimulated conditions. Hence, a conditional *Casp1^C284AFlag^* knock-in mouse model is being established.

This study was supported by the Federal Ministry of Education and Research (BMBF; Deutsches Netzwerk für Primäre Immundefekte PID-NET) and by EU Marie Curie International Reintegration grant no. GA-2007-224894 (F.P.).

## Disclosure of interest

None declared

